# Supplementary material for: Laminin Alpha 2 Enhances the Protective Effect of Exosomes on Human iPSC-Derived Cardiomyocytes in an In Vitro Ischemia-Reoxygenation Model
Source: Int J Mol Sci. 2024 Mar 28;25(7):3773. doi: 10.3390/ijms25073773 (PMC11011704; doi:10.3390/ijms25073773)
Supplement: Supplementary file 1 [file ijms-25-03773-s001.zip › ijms-2872617-supplementary.pdf]

Supplement

Laminin Alpha 2 Enhances the Protective Effect of Exosomes on Human iPSC-Derived Cardiomyocytes in an In Vitro Ischemia-Reoxygenation Model

Fernanda C. P. Mesquita [1], Madelyn King [1], Patricia Luciana da Costa Lopez [1], Shiyanth Thevasagayampillai [2], Preethi H. Gunaratne [2] and Camila Hochman-Mendez [1,*]

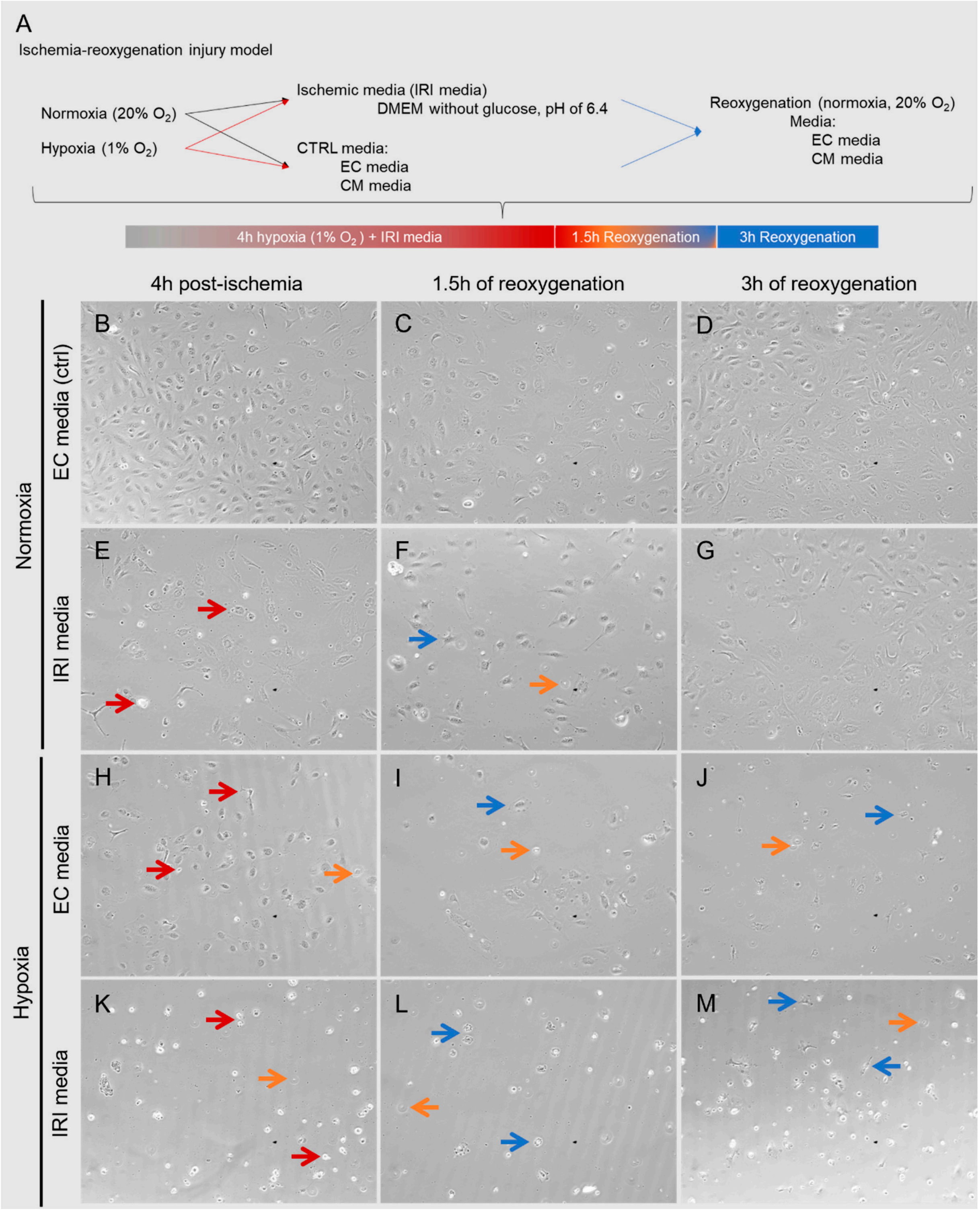

Supplemental Figure S1. Ischemia-reoxygenation in vitro model. A. Schematic diagram showing the main steps of the ischemia-reoxygenation in vitro. EC, endothelial cells; CM, cardiomyocytes. B. Brightfield images of endothelial cells under normoxia and hypoxia, and after 1.5 and 3 hours of reoxygenation. Blue arrows indicate healthy cells, orange arrows injured cells, and red arrows (floating) dead cells.

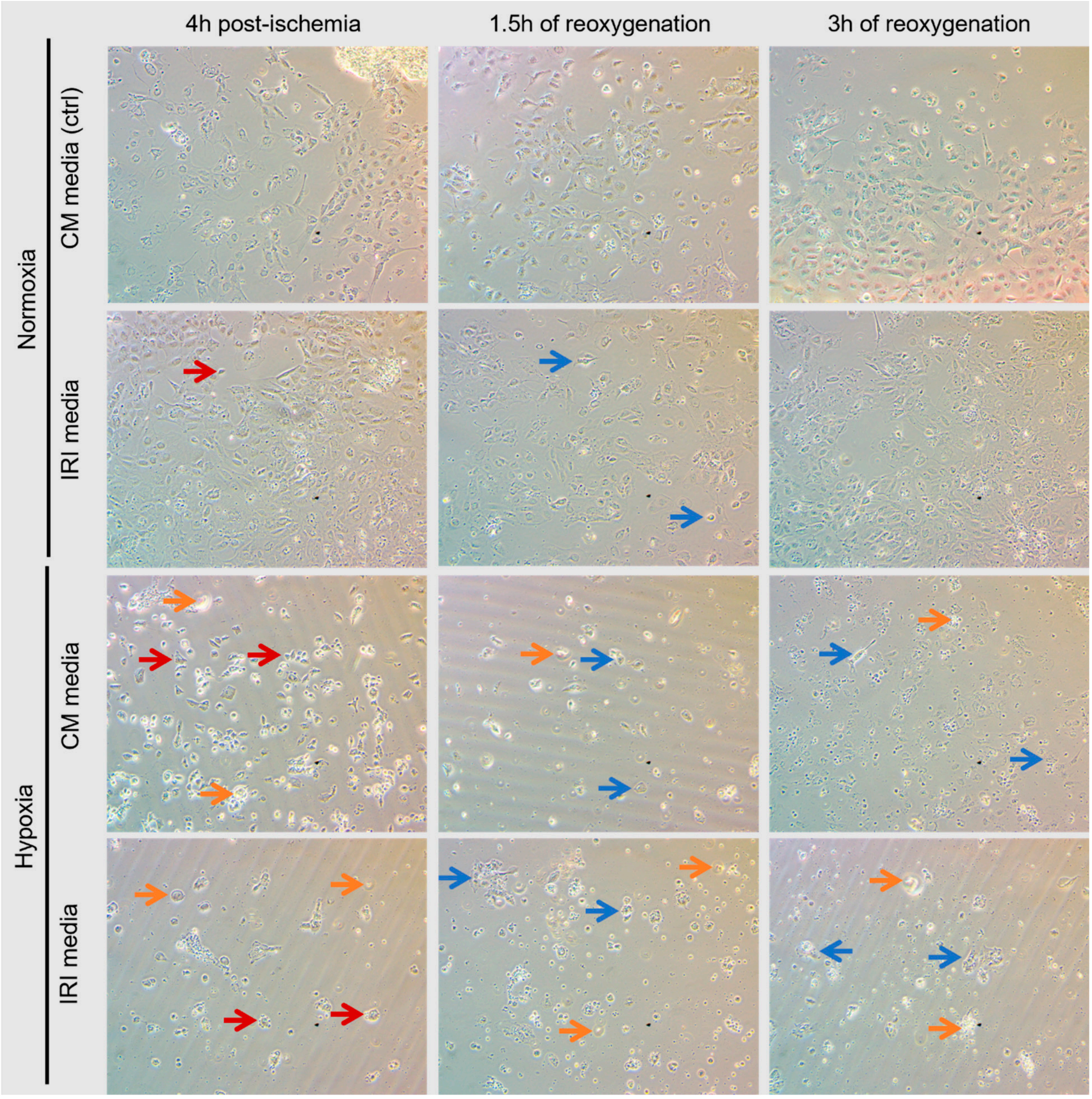

Supplemental Figure S2. Brightfield images of cardiomyocytes under normoxia and hypoxia, and after 1.5 and 3 hours of reoxygenation. Blue arrows indicate healthy cells, orange arrows indicate injured cells, and red arrows indicate (floating) dead cells.

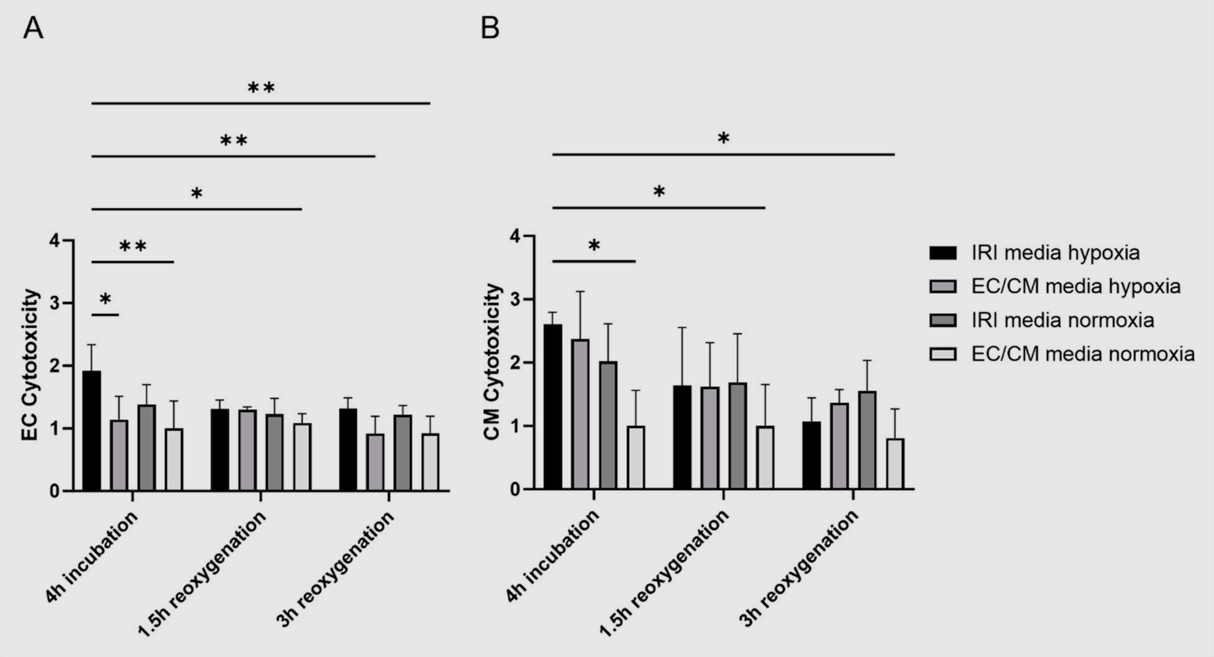

Supplemental Figure S3. Membrane integrity of endothelial cells (EC) and cardiomyocytes (CM) in an ischemia-reoxygenation model. A-B. Membrane integrity assay (LDH) indicates that ischemic medium is necessary to promote cell injury after 4 hours of hypoxia.

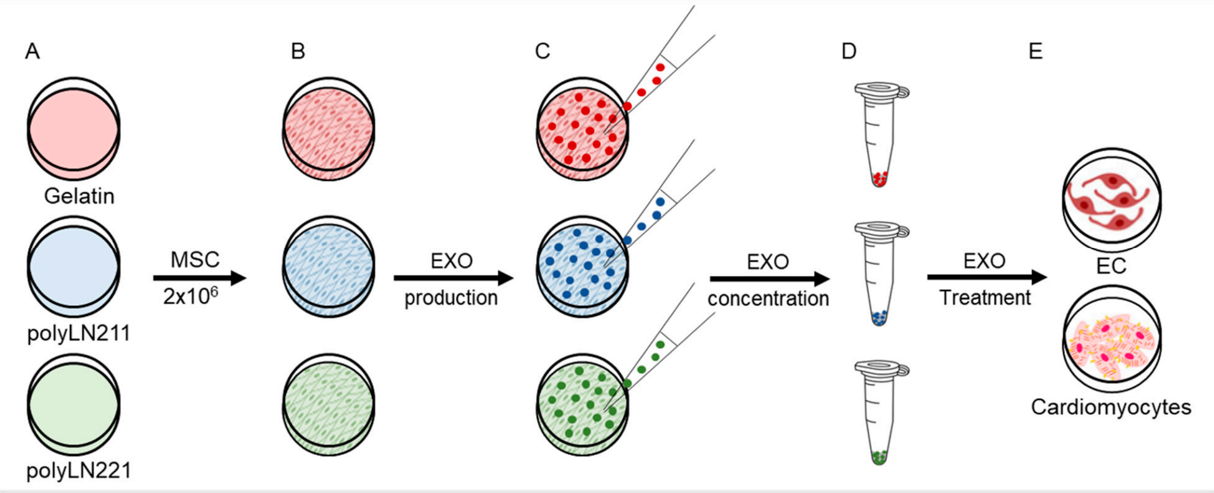

Supplemental Figure S4. Schematic diagram of EXO-LAMA2 production. A. Plates were coated with gelatin (red), polyLN211 (blue), and polyLN221 (green) overnight. MSCs were plated under different substrates (B), and EXOs were collected (C) and concentrated (D) for in vitro experiments (E).

Supplemental Table S1. List of the main unsupervised hierarchical clustering genes between Gelatin- polyLN211 and polyLN221.

Gelatin	PolyLN211	PolyLN221	Name	Function
NDUFS8			NADH:Ubiquinone Oxidoreductase Core Subunit S8	The encoded protein is involved in the binding of iron-sulfur clusters of Complex I and, as such, is required in the electron transfer process.
SLCO5A1		SLCO5A1	Solute Carrier Organic Anion Transporter Family Member 5A1	Predicted to be involved in sodium-independent organic anion transport. Located in intracellular membrane-bounded organelle and plasma membrane
LOC107987279		LOC107987279		Uncharacterized. Affiliated with the ncRNA class.
GAPDH	GAPDH		Glyceraldehyde-3-Phosphate Dehydrogenase	The product of this gene catalyzes an energy-yielding step in carbohydrate metabolism.
EEF1A1	EEF1A1		Eukaryotic Translation Elongation Factor 1 Alpha 1	Responsible for the enzymatic delivery of aminoacyl tRNAs to the ribosome.
RAB13	RAB13		Ras-Related Protein Rab-13	Regulates membrane trafficking between trans-Golgi network and recycling endosomes. The encoded protein is involved in the assembly of tight junctions. Plays a role in forming a barrier between luminal contents and the underlying tissue. Additional functions associated with the protein include endocytic recycling of occludin and regulation of epithelial cell scattering.
BCYRN1	BCYRN1		Brain Cytoplasmic RNA 1	Its product represents an example of a primate tissue-specific RNA polymerase III transcript.
FTH1	FTH1		Ferritin Heavy Chain 1	Encodes the heavy subunit of ferritin, the major intracellular iron storage protein in prokaryotes and eukaryotes. A major function of ferritin is the storage of iron in a soluble and nontoxic state.
ACTB	ACTB		Actin Beta	Involved in cell motility, structure, integrity, and intercellular signaling. The encoded protein is a major constituent of the contractile apparatus and one of the two non-muscle cytoskeletal actins that are ubiquitously expressed.
CDC27P10		CDC27P10	Cell Division Cycle 27 Pseudogene 10	Uncharacterized pseudogene
LOC107987294				Uncharacterized RNA Gene, affiliated with the ncRNA class
NDUFS7	NDUFS7	NDUFS7	NADH:Ubiquinone Oxidoreductase Core Subunit S7	Subunit of one of the complexes that forms the mitochondrial respiratory chain. This complex functions in the transfer of electrons from NADH to the respiratory chain, and ubiquinone is believed to be the immediate electron acceptor for the enzyme.
	RNR2		RNA, Ribosomal Cluster 2	Affiliated with the cluster RNA class. Among its related pathways are peptide chain elongation.
	RNR1		RNA, Ribosomal Cluster 1	Affiliated with the cluster RNA class. Among its related pathways are peptide chain elongation.
	VIM		Vimentin	Intermediate filaments, along with microtubules and actin microfilaments, make up the cytoskeleton. Responsible for maintaining

			cell shape and integrity of the cytoplasm and stabilizing cytoskeletal interactions. Involved in cell attachment, migration, and signaling.
RN7SL2	RN7SL2	RNA Component of Signal Recognition Particle 7SL2	Cytoplasmic ribonucleoprotein complex that mediates cotranslational insertion of secretory proteins into the lumen of the endoplasmic reticulum.
RN7SL1		RNA Component of Signal Recognition Particle 7SL1	Cytoplasmic ribonucleoprotein complex that mediates cotranslational insertion of secretory proteins into the lumen of the endoplasmic reticulum.
KCNA7	KCNA7	Potassium Voltage-Gated Channel Subfamily A Member 7	Expressed preferentially in skeletal muscle, heart, and kidney. Functions include regulating neurotransmitter release, heart rate, insulin secretion, neuronal excitability, epithelial electrolyte transport, smooth muscle contraction, and cell volume.
LOC107985681	LOC107985681		Uncharacterized. Affiliated with the ncRNA class.
DONSON	DONSON	DNA Replication Fork Stabilization Factor DONSON	Replisome component that maintains genome stability by protecting stalled or damaged replication forks.
MLLT3	MLLT3	Myeloid/Lymphoid Or Mixed-Lineage Leukemia Translocated To Chromosome 3 Protein	Enables chromatin binding activity and lysine-acetylated histone binding activity. Involved in several processes, including hematopoietic stem cell differentiation; positive regulation of transcription, DNA-templated; and regulation of stem cell division. Acts upstream of or within negative regulation of canonical Wnt signaling pathway and positive regulation of Wnt signaling pathway, planar cell polarity pathway.
TBP	TBP	TATA-Box Binding Protein	May participate in basal transcription, serve as coactivators, function in promoter recognition, or modify general transcription factors to facilitate complex assembly and transcription initiation.
BDH1	BDH1	3-Hydroxybutyrate Dehydrogenase (Heart, Mitochondrial)	The encoded protein catalyzes the interconversion of acetoacetate and (R)-3-hydroxybutyrate, the two major ketone bodies produced during fatty acid catabolism.
C5orf66-AS1		Esophagus Epithelial Intergenic Associated Transcript	Uncharacterized. Affiliated with the lncRNA class
LOC105374347			Uncharacterized. Affiliated with the ncRNA class.
	AR	Androgen Receptor	Ligand-activated transcription factor that regulates eukaryotic gene expression and affects cellular proliferation and differentiation in target tissues.

Supplemental Table S2. List of differentially expressed genes between polyLN211 and Gelatin with p<0.01.

Protein	Gene	Log Fold Change	Main Function
Heat Shock Protein Family A (Hsp70) Member 8	HSPA8	6.580543	Heat-shock protein, chaperone, facilitate correct folding, ATPase in the disassembly of clathrin-coated vesicles during transport of membrane components through the cell.
NEXN Antisense RNA 1*	NEXN-AS1	5.703816	Lung cancer and septic myocarditis diseases associated.
Cyclin D3	CCND3	-4.6824	Cyclins function as regulators of CDK kinases.
Glutamate Metabotropic Receptor 6	GRM6	-4.89148	G-protein coupled receptor for glutamate, triggers signaling via guanine nucleotide-binding proteins (G proteins), modulates the activity and inhibits adenylate cyclase, stimulates TRPM1 channel activity and Ca(2+) uptake.
Phosphoinositide-3-Kinase Regulatory Subunit 1	PIK3R1	-5.00752	Glucose uptake and glycogen synthesis in insulin-sensitive tissues; signaling in response to FGFR1, FGFR2, FGFR3, FGFR4, KITLG/SCF, KIT, PDGFRA, PDGFRB, ITGB2; and modulates the cellular response to ER stress.
EvC Ciliary Complex Subunit 2*	EVC2	-5.18591	Bone formation and skeletal development.
Insulin Like Growth Factor 2 Receptor	IGF2R	-6.81168	Intracellular trafficking of lysosomal enzymes, activation of transforming growth factor beta, and the degradation of insulin-like growth factor 2.
LanC Like Family Member 3*	LANCL3	-7.15292	Carbohydrate metabolic process.
MAU2 Sister Chromatid Cohesion Factor	MAU2	-7.36101	Cohesin loading and maintenance of mitotic sister chromatid cohesion.
REXO1 Like 1, Pseudogene	REXO1L1P	-7.44354	Nucleic acid binding and exonuclease activity.

*New mRNA described in human exosome according to Vesiclepedia's 2024.

Supplemental Table S3. List of differentially expressed genes between polyLN221 and Gelatin with p<0.01.

Protein	Gene	Log Fold Change	Main Function
Thymosin Beta 4 X-Linke	TMSB4X	9.061362	Actin sequestering protein and has role in regulation of actin polymerization and cell proliferation, migration, and differentiation.
Ribosomal Protein L11	RPL11	8.294152	Ribosomal component of the 60S subunit.
NME/NM23 Nucleoside Diphosphate Kinase 2	NME2	8.267342	Nucleoside diphosphate kinase; pyrimidine, and nucleotides metabolism.
Ribosomal Protein L28	RPL28	6.802163	Ribosomal component of the 60S subunit. Peptide chain elongation and rRNA processing in the nucleus and cytosol.
Ribosomal Protein L35	RPL35	6.740447	Ribosomal component of the 60S subunit. Peptide chain elongation and rRNA processing in the nucleus and cytosol.
Ras-Related Protein Rab-13	RAB13	6.029683	G proteins and plays a role in regulating membrane trafficking between trans-Golgi network and recycling endosomes; assembly of tight junctions, which are components of the apical junctional complex of epithelial cells; the complex plays a role in forming a barrier between luminal contents and the underlying tissue. Endocytic recycling of occludin, regulation of epithelial cell scattering, neuronal regeneration, and regulation of neurite outgrowth.
Thymosin Beta-10	TMSB10	5.64525	Predicted to enable actin monomer binding activity, regulation of cell migration and sequestering of actin monomers. Predicted to be located in cytoskeleton and to be active in cytoplasm, VEGFA-VEGFR2 signaling and Extrafollicular and follicular B cell activation by SARS-CoV-2.
Metallothionein-2	MT2A	5.502487	Bind divalent heavy metal ions, altering the intracellular concentration of heavy metals in the cell. Act as antioxidants, protect against hydroxyl free radicals, and are important in homeostatic control of metal in the cell. Role in detoxification of heavy metals. Controls intracellular zinc levels, affecting apoptotic and autophagy pathways. Related pathways are Metal ion SLC transporters and interferon gamma signaling.
Elongation Factor 1-Gamma	EEF1G	5.276311	Enzymatic delivery of aminoacyl tRNAs to the ribosome, may be involved in regulating the assembly of multisubunit complexes containing this elongation factor and aminoacyl-tRNA synthetases. Related pathways are Signaling by ALK in cancer and Peptide chain elongation.
Calmodulin 1	CALM1	5.073639	Calcium-induced activation of calmodulin regulates and modulates the function of cardiac ion channels.
Trafficking Kinesin Protein 2	TRAK2	5.042845	Predicted to enable GABA receptor binding activity and myosin binding activity, mitochondrion distribution; organelle transport along microtubule; and protein targeting. Related pathways Miro GTPase Cycle and Signaling by Rho GTPases.

Microtubule Crosslinking Factor 1*	MTCL1	4.938853	Enables microtubule binding activity. Predicted to be involved in establishment or maintenance of epithelial cell apical/basal polarity; microtubule bundle formation; and positive regulation of protein targeting to membrane.
Kinesin Family Member 1C	KIF1C	4.561988	Member of the kinesin-like protein family- microtubule-dependent molecular motors that transport organelles within cells and move chromosomes during cell division. Related pathways are Golgi-to-ER retrograde transport and Vesicle-mediated transport.
Exocrine Differentiation And Proliferation Factor	PPDPF	4.298254	Predicted to be involved in cell differentiation.
FAT Atypical Cadherin 3*	FAT3	-4.77278	Predicted to enable calcium ion binding activity, cell-cell adhesion, negative regulation of dendrite development; neuron migration; and retina layer formation, integral component of membrane.
Insulin Like Growth Factor 2 Receptor	IGF2R	-4.78492	Receptor for both insulin-like growth factor 2 and mannose 6-phosphate. Related pathways are Apoptotic Pathways in Synovial Fibroblasts and Innate Immune System.
Asialoglycoprotein Receptor 2*	ASGR2	-4.81463	Transmembrane receptor protein that plays a critical role in serum glycoprotein homeostasis by mediating the endocytosis and lysosomal degradation of glycoproteins with exposed terminal galactose or N-acetyl galactosamine residues. May facilitate hepatic infection by multiple viruses including hepatitis B and is also a target for liver-specific drug delivery. Related pathways are Metabolism of proteins and Transport to the Golgi and subsequent modification.
Phosphoinositide-3-Kinase Regulatory Subunit 1	PIK3R1	-4.89496	Phosphorylates the inositol ring of phosphatidylinositol, role in the metabolic actions of insulin. Related pathways are Prolactin Signaling and IL-9 Signaling Pathways.
Forkhead Box K1	FOXK1	-5.25328	Enables 14-3-3 protein binding activity; DNA-binding transcription repressor activity, RNA polymerase II-specific; and transcription cis-regulatory region binding activity. Involved in several processes, including cellular glucose homeostasis; negative regulation of autophagy; and regulation of transcription, DNA-templated. Related pathways are Metabolism of proteins and Deubiquitination.
EvC Ciliary Complex Subunit 2*	EVC2	-5.60179	Functions in bone formation and skeletal development.
MAU2 sister chromatid cohesion factor	MAU2	-6.06964	Enables protein N-terminus binding activity. Involved in cohesin loading and maintenance of mitotic sister chromatid cohesion. Part of Scc2-Scc4 cohesin loading complex.
Uncharacterized	LOC107986445	-7.41473	-
Cytochrome C Oxidase Assembly Factor 5	COA5	-7.47343	Ortholog of yeast Pet191, which in yeast is a subunit of a large oligomeric complex associated with the mitochondrial inner membrane and required for the assembly of the cytochrome c oxidase complex.

Glycoprotein Nmb	GPNMB	-7.50249	Type I transmembrane glycoprotein that shows homology to the pMEL17 precursor, a melanocyte-specific protein. GPNMB may be involved in growth delay and reduction of metastatic potential.
Mitogen-Activated Protein Kinase Binding Protein 1	MAPKBP1	-7.52437	Scaffold protein that regulates the JNK (c-Jun N-terminal kinase) and NOD2 (nucleotide-binding oligomerization domain-containing protein 2) signaling pathways.
Versican	VCAN	-7.61094	Member of the aggrecan/versican proteoglycan family, a large chondroitin sulfate proteoglycan and is a major component of the extracellular matrix. Related in cell adhesion, proliferation, proliferation, migration, and angiogenesis and plays a central role in tissue morphogenesis and maintenance.
Heparan Sulfate D-Glucosaminyl 3-O-Sulfotransferase 1	HS3ST1	-7.63847	Enzymes are key components in generating a myriad of distinct heparan sulfate fine structures that carry out multiple biologic activities.
Indian Hedgehog Signaling Molecule	IHH	-7.64507	Member of the hedgehog family of proteins, generate multiple protein products, including an N-terminal fragment that is involved in signaling. Essential for secreted signaling molecules that regulate a variety of developmental processes including growth, patterning, and morphogenesis. The protein encoded by this gene specifically plays a role in bone growth and differentiation.
Uncharacterized	LOC105370567	-7.73373	-
LanC-Like Protein 3 *	LANCL3	-7.76518	Predicted to be involved in carbohydrate metabolic process.
Uncharacterized	LOC100130207	-7.88387	-
Cell Division Cycle 27 Pseudogene 9*	CDC27P9	-8.12456	Pseudogene

*New mRNA described in human exosome according to Vesiclepedia's 2024.

Supplemental Table S4. List of differentially expressed genes between polyLN211 and polyLN221 with p<0.01.

Protein	Gene	Log Fold Change	Main Function
SH3 domain binding glutamate rich protein like 3	SH3BGRL3	7.553577	Nuclear body.
acidic nuclear phosphoprotein 32 family member B	ANP32B	8.077543	Enables RNA polymerase binding activity and histone binding activity, apoptosis; nucleosome assembly; and positive regulation of protein export from nucleus.
tropomyosin 2	TPM2	8.073332	Member of the actin filament binding protein family, and mainly expressed in slow, type 1 muscle fibers.
ribosomal protein S19	RPS19	7.870294	Component of the 40S ribosomal subunit; extra-ribosomal function in differentiation and proliferation.
ribosomal protein L12	RPL12	7.683415	Ribosomal protein that is a component of the 60S subunit.
tropomyosin 4	TPM4	7.419697	Actin-binding proteins involved in the contractile system of striated and smooth muscles and the cytoskeleton of non-muscle cells. Tropomyosins are dimers of coiled-coil proteins that polymerize end-to-end along the major groove in most actin filaments.
endothelial differentiation related factor 1	EDF1	7.324753	Regulate endothelial cell differentiation, lipid metabolism, and hormone-induced cardiomyocyte hypertrophy.
pancreatic progenitor cell differentiation and proliferation factor	PPDPF	7.286403	Predicted to be involved in cell differentiation.
ribosomal protein S16	RPS16	7.229725	Ribosomal component of the 40S subunit. The protein belongs to the S9P family of ribosomal proteins.
major histocompatibility complex, class II, DR beta 1	HLA-DRB1	7.018186	Immune system by presenting peptides derived from extracellular proteins.
trafficking kinesin protein 2	TRAK2	6.708681	Predicted to enable GABA receptor binding activity and myosin binding activity; active in cytoplasmic vesicle; dendrite; and mitochondrion.
ribosomal protein L13a	RPL13A	6.572105	Component of the 60S subunit; repression of inflammatory genes as a component of the IFN-gamma-activated inhibitor of translation complex.
metallothionein 2A	MT2A	5.665103	Bind divalent heavy metal ions, altering the intracellular concentration of heavy metals in the cell; antioxidants, protect against hydroxyl free radicals, important in homeostatic control of metal in the cell, and play a role in detoxification of heavy metals.

S100 calcium binding protein A6	S100A6	5.647878	2 EF-hand calcium-binding motifs; may function in stimulation of Ca2+-dependent insulin release, stimulation of prolactin secretion, and exocytosis.
galectin 1	LGALS1	5.186484	Member of beta-galactoside-binding proteins family, modulation of cell-cell and cell-matrix interactions. This gene product may act as an autocrine negative growth factor that regulates cell proliferation.
ANTXR cell adhesion molecule 1	ANTXR1	5.005585	Type I transmembrane protein, tumor-specific endothelial marker for colorectal cancer; receptor for *Bacillus anthracis* toxin.
ribosomal protein L39	RPL39	4.648474	Component of the 60S subunit.
ferritin light chain	FTL	4.524936	Major intracellular iron storage protein, iron uptake and release in different tissues.
RNA component of signal recognition particle 7SL2*	RN7SL2	4.376611	The signal recognition particle is a cytoplasmic ribonucleoprotein complex that mediates cotranslational insertion of secretory proteins into the lumen of the endoplasmic reticulum.
Cell Division Cycle 27 Pseudogene 9*	CDC27P9	-9.08347	-

*New mRNA described in human exosome according to Vesiclepedia’s 2024.
